# Perspective of Teachers and Students towards the Education Process during COVID-19 in Romanian Universities

**DOI:** 10.3390/ijerph19063409

**Published:** 2022-03-14

**Authors:** Andreea Barbu, Mirona Ana Maria Popescu, Georgiana Moiceanu

**Affiliations:** Faculty of Entrepreneurship, Business Engineering and Management, University POLITEHNICA of Bucharest, 060042 Bucharest, Romania; andreea.barbu2901@upb.ro (A.B.); mirona.popescu@upb.ro (M.A.M.P.)

**Keywords:** educational process, online teaching, educational platform, e-learning, COVID-19 impact, digital transition

## Abstract

The COVID-19 pandemic has created important changes in all areas, highlighting many vulnerabilities, but also opportunities based on the use of technology. This paper aims to provide an overview of the online educational process from two perspectives—that of students and that of professors from Romanian universities. Data were collected from 844 students from Romanian universities disregarding the area of study. To achieve the main goal of this paper, both qualitative (in-depth interviews) and quantitative methods (surveys) were used, the data being processed using the SPSS Statistical software. The results of this paper highlight the discrepancy between the perspectives of the two parties directly involved in the university educational process. The study shows that the pandemic forced both stakeholders to work harder than before, which negatively affected the way the educational process unfolded, the pleasure of the teaching/learning process, the level of enthusiasm, and sometimes even the academic results. The final conclusions of this paper also highlight the need to make financial investments for the acquisition of licenses to create virtual animations or simulations, as well as for training teachers in their use. Research also indicates that to maintain students’ attention in class, especially online, teachers should use new teaching strategies, such as the use of debates and brainstorming sessions.

## 1. Introduction

There is no denying the fact that the COVID-19 pandemic brought massive disruption in all fields of activity, and given the circumstances, the traditional ways of teaching and learning had to evolve to cope with the situation. Given the high risk of the COVID-19 spread, all institutions had to block physical face-to-face activities, and this had a huge impact on educational universities.

Ever since the beginning of the pandemic, and still being an ongoing process, the main objective for all countries is to decrease the raid spread of SARS-CoV-2 [1]. New trends in society and the emergence of new technologies in the dawn of the fourth industrial revolution come with both benefits and challenges [2]. Educational systems around the world quickly adopted e-learning platforms, to deliver the process of teaching [3].

The process of e-learning is the concept of delivering education via the internet, among all learners involved [4]. Online education is flexible and cost-effective, allowing access to learning resources [5]. Digitalization is not a new thing for universities, most of them already had educational online platforms implemented before COVID-19, but they were never meant to be the sole channel of delivering knowledge [6].

Although a good concept on paper, online learning continues to be a challenge for developing countries due to a lack of resources, like digital devices, or lack of reliable and fast internet connection, mainly in rural areas [7]. The student adoption level of online learning is also heavily influenced by parent perception of this technology. If parents see the benefits of online learning, they will influence the students into positively adapting and using this new way of learning [8].

Wanga et al. [9] conducted a study outlining the positive and negative aspects of e-learning, to evaluate student level of satisfaction, and their results produced mixed results. While the students were satisfied by the overall education experience, they complained about the quality of internet connection and the devices they were using.

A recent study conducted by Karangul et al. [10] aimed to explore the digital literacy of different school levels, ages, and gender. Their results showed no significant differences between the variables, all groups showing medium to high digital literacy levels. The reason for this, pointed out by the authors, is that students were already heavily invested in information technology before the COVID-19 pandemic, and e-learning was a near-perfect fit for them.

Filho et al. [11] have outlined the challenge of quickly coping with e-learning, for teachers and students alike. Their paper also mentioned concerns about teaching effectiveness using this method, the lack of personal communication, and questioned student motivation. 

Following an online survey at the University of Petrosani, with the purpose to monitor the online learning implementation and satisfaction levels, Edelhauser et al. [12] showed that students quickly adapted to this new way of learning, but pointed out that using different online delivery tools had its downside, concluding that online learning is a great fit for Romanian education if using a centralized platform for online courses.

In their paper, Toader et al. [13] saw the benefits of digitalization on tertiary education, but also for sustainable development, while pointing out the need for further investments in digitalization and implementation of new technologies in Romanian universities. The students interviewed during their survey reported a state of pressing isolation due to a lack of interaction given by the social distancing protocol.

A reflection on current Romanian Universities activity during the COVID-19 pandemic was also published by Marchis [14], stating that the pandemic, unfortunately, moves faster than decision-makers and that digitalization should have been implemented regardless of current circumstances, and not reactively. The author also stated that information is interactive now, redesigning the relations between people, and between people and information, dissolving spatial, temporal, and financial barriers in the path of knowledge.

In a recent study published by Molea et al. [15], a few weaknesses of Romanian online learning were researched, concluding that the barrier between the student and the teacher due to the participation with the webcam stopped, and sound disturbances given by poor connection or background noise. 

While most of the studies concerning e-learning in the current pandemic focused on student satisfaction levels, few studies have been made for the Romanian students’ transition phase to online education.

During their research, Edu et al. [16] found out that even though Romania has excellent internet speed and coverage, the transition in educational institutions towards online learning is still limited, and Romanian students reported that the lack of interaction was and is an important issue of discomfort, because the online environment, no matter how well it is constructed, cannot replace face-to-face interactions. This was also pointed out by Dumford et al. [17] and concluded that students engaged in online education have lower interaction levels and do not participate in collaborative learning, leading to a lack of social skill development.

In their paper, Catana et al. [18] designed and tested a theoretical model for explaining the influence of technology, individual involvement, psychological well-being, household activities, and physical inactivity on Romanian student online learning activities, and their results showed that students were negatively influenced by the technology factor and physical inactivity while being positively influenced by the psychological well-being factor. Their results also showed that students prefer traditional face-to-face learning, while teachers stated that a hybrid system between traditional and online learning is the way to go in the future.

Considering the results of the research and the way the education system still tried to cope with the changes COVID-19 in all its forms bring to the educational system overall, we conducted an overview of the educational process during COVID-19 in higher educational institutions, considering the perception of both parties involved, student and university teachers. The topic of the paper is the result of the authors experience to cope with the changes that COVID-19 pandemic brought to higher educational system and the result of the discussions with students that took place during class hours. Considering that some differences in opinions were observed, the authors decided to conduct a study in which the perceptions from both parties are taken into consideration, even compared. From the discussions some directions of research could be drown, while others were added by the authors to cover more aspects. 

Therefore, following the results of researchers towards higher education in the offset of the pandemic COVID-19 and the reasons mentioned, the authors of this paper propose the following five directions of research reached by developing an online questionnaire:

1. The most used educational platform in online teaching;
2. The most used strategies in online teaching;
3. How do teachers and students evaluate individual and teamwork;
4. The daily average time spent before and after the pandemic COVID-19 to preparation/learning process;
5. Perception of both teachers and students towards e-learning.

Getting the answer for these directions will help in bringing additional information regarding the impact of the COVID-19 pandemic on the higher educational system. Furthermore, at the end of the pandemic, the authorities assessing the overall changes in this domain can gather data from the research done by scientists, thus our work can be a contribution to this. The perspective from both parties will supply data valuable to shift the way educational process takes place considering the needs and capabilities. 

## 2. State of the Art

The digitalization and the Internet have changed the education system all over the world. However, the COVID-19 pandemic situation had contributed to the process of learning differently from one country to another, based also on the environment. Most of the human interactivity has been shifted to digital media and online learning methods.

### 2.1. Outcomes of COVID-19 in Higher Education Institutions from Underdeveloped Countries

A study conducted in Pakistan, an underdeveloped country, showed that most of the students encountered technical and monetary difficulties, which led to unsatisfying results [19]. 

In Lahore [20], a qualitative case study highlighted the advantages of online learning and its limitations. As a result, student-centered learning was encouraged as well as lessons with reduced cognitive load, but higher interactivity. In Bangalore city [21], teachers and students’ perception towards the improvement of an online learning process indicates that quality and timely interaction, technical support availability, and structured lessons establish satisfaction to both parts. 

In Indonesia [22], according to quantitative research, students that have knowledge of MS Office and technical skills adapted quicker to online lessons. The success of this type of lesson is given by participants and resources, but it comes also with negative outcomes like lack of social interaction, internet access, and discipline. Advantages, constraints [23], and solutions were also investigated using a survey in private universities in this country. The time and space barriers were eliminated by adopting online learning, but the instability of Wi-Fi affected the transmission of the lessons; therefore, the students’ concentration was reduced. At the University Megarezky in Indonesia [24], students saw the advantages of online learning in terms of flexibility, which triggered their interest. 

Moreover, according to them, WhatsApp is the best application used to communicate lessons and messages for both teachers and students. In another scientific paper [25], even if flexibility came again as a strength, most of the students encountered difficulties in conversation, issues regarding unstable internet connection, which led to lower motivation. A total of 91% of respondents agree that face-to-face learning and supporting tools are a must for online learning along with applications and stability of the internet.

Qualitative research [26] was carried out in Indonesia to establish the psychological impacts of e-learning on students. The output highlighted that after a period of time their mood has changed due to many assignments, considerable anxiety was noted, and students got bored. As a result of the study, measures regarding the psychological well-being of students must be implemented by putting them in touch with certified experts in psychology.

The students’ perception from the top 10 universities in Surabaya [27] to online learning in the offset of COVID-19 was analyzed using the theory of Kenneth K., Edward M., Judy C. Pearson, and Paul E. Nelson (2008) and the official social media accounts. The respondents agreed that online learning was the proper solution to be implemented in this context, but they were not satisfied with the facilities provided by the universities in terms of the internet and the high degree of assignments.

In Ghana [28], even if platforms like the UCC Moodle platform, Alison, and Google classroom were known, online learning was negatively perceived because students want formal orientation along with training. Another issue concerns the high costs of internet usage, which many families are not able to sustain. Universities should obtain lower costs for their enrolled students, and provide better instructional support and flexibility when it comes to assignments deadlines because of poor Wi-Fi connection.

Students from Universitas Islam Raden Rahmat (Unira) Malang participated in a study [29] conducted online via Google Form. The data collected pointed out their negative position towards online learning due to a lack of proper planning, as well as enormous assignments which led to a lower involvement.

In Malaysia [30], a case study was conducted through qualitative research to establish the challenges in higher education: students’ attention to lessons in the online environment, the level of satisfaction towards platforms used, learning tools such as books were less used, internet access, class attendance. 

### 2.2. Outcomes of COVID-19 in Higher Education Institutions from Developed Countries

In Saudi Arabia [31], many systems and platforms were used to replace face-to-face interaction to protect the spread of the virus in universities. All these changes should be noted in portfolios to establish the strengths and weaknesses of technology from an educational point of view. The way of teaching in an online environment [32] had an impact on the level of stress among students: their biggest fear is related to the final semester exams and assignments. 

The advantages and disadvantages of online learning on the current offset were also studied through qualitative research on undergraduate students from Abu Dhabi [33]. This type of learning proved to be cost and time efficient, giving the students the possibility to improve their participation. On the other side, there are problems like workload, the ability to stay focused, and technical issues. Therefore, educational institutions should invest a part of their capital in adopting measures that may overcome emergency situations such as the COVID-19 pandemic.

According to a study in Poland [34], e-learning was the core method of teaching and the survey applied to a sample of 804 medical students who stated that the main advantage is given by the ability to stay home, followed by access to online materials any time of the day and having your own comfortable surroundings. There was no difference in the ability of learning; however, e-learning is less effective in terms of increasing skills and social competencies.

The adaptability of students in Israel to the sudden shift of traditional face-to-face learning to online learning was investigated [35]. A comparison between these two methods based on the data collected determined that their position towards the online environment is negative. Another conclusion was related to the adaptability to online learning based on each student’s personality.

A survey was applied to medical students from the Philippines [36], a developing country, to find out the barriers brought by online learning. Even if most of them have mobile telephones, computers, and paid subscriptions to the internet, 41% were capable to engage in online learning from a psychical and mental perspective. The barriers were systematized into five categories: technological, individual, domestic, institutional, and community barriers. These are given by each student’s learning style, the chores that they must perform at home, and the low degree of communication with teachers.

In China [37], using a difference-in-differences approach, students who benefit from a computer or laptop fared better than the ones who have a mobile phone. The online environment was beneficial in terms of exam results for all students except the very best ones.

The International Association of Universities conducted a study in 2020 to analyze the outcomes of COVID-19 on universities and other higher education institutions (HEI) from all five continents [38]. A total of 59% of HEIs have stopped campus activities and closed institutions completely, but 91% have infrastructure to continue their activity online, and 48% were supported by their government to complete the academic year. The pandemic-caused offset created new opportunities in terms of partnership for 31% of respondents and more flexible learning possibilities. Mainly, the problems from shifting from traditional to digital were due to technical infrastructure, competences for distance learning, and requirements of specific fields of study.

The concerns of 644 college students from the US, Asia, and Europe [39] were translated and indexed by theme to support the continuity of learning as follows: education (188 students), safety (183 students), mental health (113 students), employment/finances (105 students), future uncertainty (96 students), and relationships (80 students). To support their healthy learning environment, strong systems for managing communications and the flow of information are needed, as well as virtual communities and national organizations that combat stigma as National Alliance on Mental Illness and Worlds Psychiatric Association in Canada, Europe, and Oceania.

A study carried out in 27 countries of the European Union [40] presented that worldwide HEI were forced to find solutions quickly to adapt to remote education, and their main focus was on technical issues rather than pedagogical methodologies for distance learning, resulting in a psychological impact for both students and teachers.

The mental health concern was raised in another USA HEI study [41], involving 195 students. A total of 71% of them encountered stress and anxiety issues. Among the multiple stressors were fear for their health and their loved ones (91%), lack of concentration (89%), disrupting sleep (86%), and decreased social interactions (82%).

## 3. Materials and Methods

Before collecting the data, the authors went through several phases of work. For a start, the existing statistical data were consulted on specialized sites such as Eurostat and Statista, which present various statistical aspects of the educational process carried out during the pandemic. The Scopus, Web of Science, and Springer databases were then searched to analyze the educational process described nationally and internationally by the academic community. Keywords such as “educational process”, “online teaching”, “educational platform”, “e-learning”, “COVID-19 impact”, and “digital transition” were used to filter the search results, with articles published in the period 2020–2022 that described the educational process carried out during the COVID-19 pandemic. The most relevant articles for the searches performed in terms of the keywords used, as well as the number of citations, were then analyzed by the authors. Based on the literature review, two specific groups (students and teachers) were identified as the key internal stakeholders. 

Thus, the authors targeted 2 separate groups of study: one consisting of Romanian students and one consisting of teachers from Romanian universities. The authors did not limit their study to analyze the situation of a single university, because they wanted to collect data that would be as representative as possible for the online education system during the pandemic, as each university was able to adapt its teaching style both according to the instructions received from the government and according to its own management directives. The fourth phase of the study consisted of selecting the right research methods to accomplish the objectives of this paper. Thus, the authors used mixed methods research [42,43], selecting qualitative (in-depth interviews), and quantitative (surveys) methods. The four mentioned phases of work were conducted between March and May 2021. 

During this period, 10 in-depth interviews were also conducted through online interviews on the Skype, Zoom, and Microsoft Teams platforms with people representing the two groups to identify the main themes of the questionnaires. The interviews were semi-structured, lasted around 35 min, and covered several research directions, such as educational platforms and electronic devices used in online education, effort made in the educational process during the pandemic and the difficulties encountered in this regard, obtained results, attractive teaching strategies, and other aspects related to online educational processes.

Based on these in-depth interviews, the authors developed 2 questionnaires, which were covering the most discussed directions of the interviews, and questions being adapted for the 2 groups of study (teachers and students).

The questionnaires were sent online to at least 5 professors from the 55 universities in Romania [44], who were asked to distribute the 2 questionnaires among their colleagues and among their students. In addition, the questionnaires for students were posted on the social media groups of the 55 universities in Romania. The data collection period was from 1 to 31 May 2021. The survey participation in this study was voluntary. 

After receiving, centralizing, and systematizing the data gathered online, the authors registered 876 responses from students and 126 from university teachers from Romania. Unfortunately, 32 out of 876 questionnaires from students were incomplete, so the final sample of students consisted of 844 people.

Of the 126 university teachers, 41.26% were males and 58.74% were females, most of the respondents having the didactic function of lecturer. In this research, most of the teachers were working in faculties with technical profiles (61.9%), while the others were working in the economic field (12.7%), medical field (7.9%), law domain (7.9%), the field of construction engineering (6.3%), or other areas (3.2%). Of the 844 students, 60.2% of them were males, most of them between 20 and 22 years of age (42.7%). A large group of the students’ sample was represented by those who were studying in technical universities (66.4%), followed by those in the economic universities (14%), while the rest were studying in universities with profiles like construction, medicine, or other types (Table 1).

## 4. Results

### 4.1. Basic Information about the Online Teaching Process in Romanian Universities

In the case of higher education, the presence of students in classes was decided by each institution based on their autonomy. The general rules of COVID-19 regarding general distance, mask-wearing, or other elements applied also in higher education, but still, there are some areas where the universities decide for themselves. Thus, starting from March 2020, most of the universities started to operate in the online environment, following the recommendation of the Minister of Education [45]. 

Before the pandemic situation, approximately half of the teachers highlighted the fact that they were using educational platforms in a small or even very small extent, while just 4.8% of them were using them to a very large extent (Table 2). 

**Figure 1:**
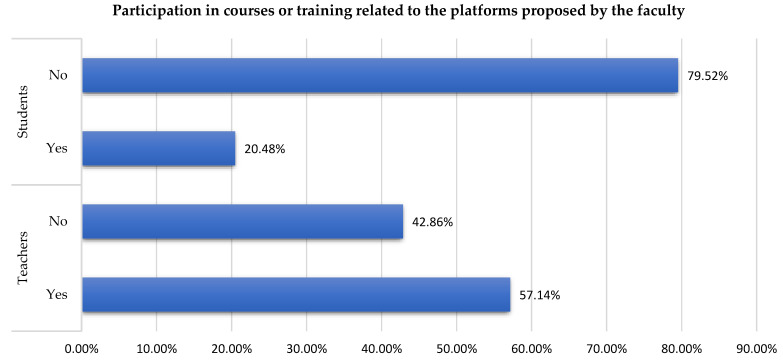
Participation in courses or training related to the platforms proposed by the faculty.

Therefore, for a part of the respondents, the pandemic situation put the students or the teachers in the situation of using certain online teaching platforms for the first time. In this regard, universities should have been preoccupied with offering courses or training to explain how these platforms can be used. Unfortunately, for various reasons, not all the educational stakeholders were participating in these courses. Thus, only 20.48% of the students and 57.14% of the teachers from this study were participating in those courses (Table 3 Figure 1).

Because most work platforms have an English language interface, the authors analyzed whether the level of English language skills influenced the participation in training platforms for the use of educational platforms (Table 3).

On the one hand, in the case of the teachers, an association between participation in courses or trainings related to the use of online platforms and the level of English language was not observed, χ^2^(5) = 10.836, *p* = 0.055 (Table 4). On the other hand, in the case of the students, an association between participation in courses or trainings related to the use of online platforms and the level of English language was observed, χ^2^(5) = 18.873, *p* = 0.002, the Cramer’s V value being 0.151 (*p* = 0.02).

Furthermore, the level of participation in courses or trainings related to the use of online platforms is associated with the level of digital skills, χ^2^(3) = 10.115, *p* = 0.018 (Table 5), the Cramer’s V value being 0.110 (*p* = 0.018).

**Figure 2:**
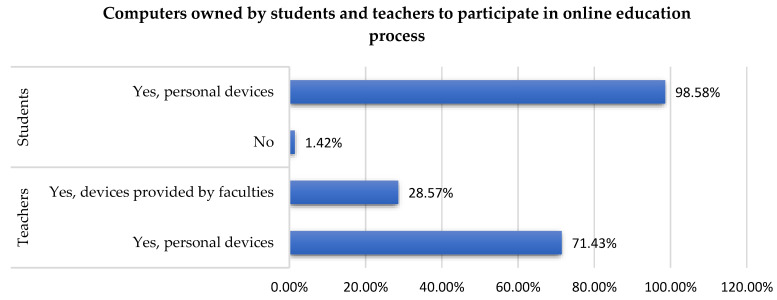
Computers owned by students and teachers to participate in online education.

All the respondents were participating in online classes at the time of the study. On one hand, 71.4% of the teachers were owning their personal electronic devices for online classes, only 28.6% of them were using laptops provided by universities (Figure 2). On the other hand, 98.6% of the students were using their personal laptops or desktops for online classes, 1.4% of them having only their smartphones for the online teaching process.

Despite this fact, it seems that only 78.2% of the students were using desktops or laptops for participating in online classes, 0.9% of them were using tablets, while a worrying percentage of 20.9 were connected online via their smartphone (Table 6). In the comment sections, some of the students that were mentioned in the last part, mentioned that they were connected online via their smartphones because they were having other priorities for those moments, or they were not so interested in what was taught to them.

From the analysis of the answers of the two analyzed groups, it is found that the most popular educational platform used in online education is Microsoft Teams, followed by other platforms such as Moodle or Google Classroom (Table 7).

### 4.2. Average Daily Duration Allocated before and after the Pandemic to the Preparation/Teaching Process, after the End of Online Classes

By using the analysis of Pearson coefficients, it seems that there are significant positive correlations between the average daily duration allocated before and after the pandemic to the teaching process, after the end of online classes, in both the cases of students and teachers (Table 8). In the students’ case, the correlation is moderate (r = 0.439, *p* < 0.01), while in teachers’ case, the correlation is weak (r = 0.338, *p* < 00.1), those results indicating that people who previously paid more attention to the teaching process after its ending, now pay even more attention to this aspect, investing more time in this direction.

On a more detailed analysis of the time invested in this direction, it is found that both teachers and students have begun to allocate more time to the online learning process (Table 9). Before COVID-19, just 6.4% of the students were investing more than 4 h for personal study, after COVID-19, the percentage of students that were spending more than 4 h for individual study rose to 41.5%. This highlights the fact that students either feel the need to work extra because they do not understand too much during online classes or receive a lot of homework and projects from teachers and are forced to devote much more time to the educational process than before, which becomes tiring and inefficient for them.

The same can be said about teachers, where their tendency to work much more than before online classes is observed. Before the pandemic, more than 90% of teachers spent less than 4 h preparing teaching materials, now 25.2% of teachers spent even more than 4 h preparing materials for courses or seminars, respectively correcting assignments. In the comments section, teachers pointed out that it is becoming more and more tiring to work so long on preparing online classes, after having to hold online classes or seminars before these classes. They complain about the headaches, but also about the back pains that they started to feel, especially in the last year. In addition, many of them report misunderstandings in the family that go from the too much time they spend on the computer after completing the work schedule, ignoring their personal life or household activities.

### 4.3. Using the Faculty Website before and during the COVID-19 Pandemic 

Regarding the consultation of the official website of the faculty to be up to date with the latest information about the education act or the educational situation (Table 10), it was found that those who used the faculty’s website before the pandemic do so now, especially teachers (r = 425, *p* < 0.01). 

The very weak, positive correlation observed in the case of students (r = 0.163, *p* < 0.01) can be explained by the fact that students can more easily find news by other means (via group leaders or social media groups), while teachers rely on finding information in a more formal setting.

### 4.4. The Most Efficient Way Students Work

**Figure 3:**
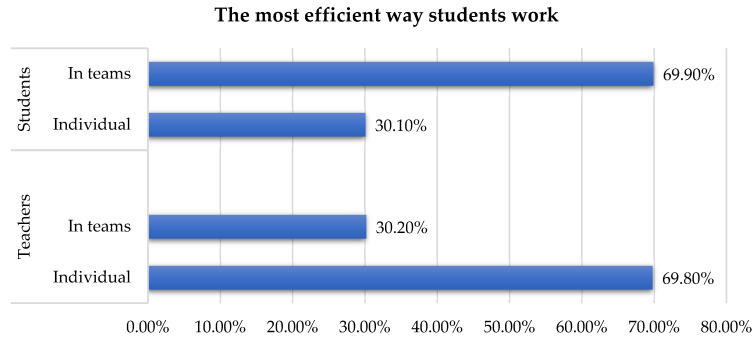
The most efficient way students work.

Regarding the way students work during the online process, it seems that there is a perception gap between how students work efficiently and how teachers consider that efficiency not to be enough to better understand the taught concepts (Figure 3).

Thus, it is found that teachers have a completely wrong impression about the preferred way of working of students, but also about their interaction needs during this period. 

### 4.5. Identifying the Most Difficult Disciplines to Be Studied Online

The most difficult disciplines to be studied online are those that involve exact mathematical and technical sciences (Table 11). Moreover, both parties claim that subjects involving practical activities and presence in laboratories are also difficult to achieve online. About 11% of teachers mentioned that classes related to the medical field are difficult to teach in the online environment, while 7% of students mentioned that all subjects can be easily transposed into the online environment.

There are both teachers and students who considered that all subjects are difficult to teach online, with the physical component of interaction being the key element missing.

### 4.6. Identifying the Most Useful Online Teaching Strategies

Both teachers and students claim that video animations and virtual simulations would be the most appropriate teaching strategies in the online environment, followed by the use of contests and attention to detail tests (Table 12). Besides these aspects, the following strategies are also important: the debates and brainstorming sessions, the projects carried out in the team, as well as the use of platforms or tools to monitor in real-time the progress or the activity performed by the students. In addition, students believe that teachers should use graphics tablets to write in real-time with them, not just talk, provide more practical examples, use graphical diagrams, and provide more summaries so that the subject could be easier for students to remember.

However, 12.5% of students stated that returning to the classical teaching system would be the best teaching strategy, as the teaching process is not effective in the online environment.

### 4.7. The Way Online Classes Are Perceived

Regarding the way in which online classes are perceived by the two parties directly involved in the educational teaching-learning process (Table 13), it is found that for students, classes are in principle tiring (49.8%), boring (28.4%), and impersonal (18.2%). 

For teachers, classes are basically impersonal (38.1%) and tiring (25.4%). However, there is also a significant share of the two parties who consider online classes to be tedious, engaging, and ingenious, while 3.2% of teachers consider the classes to be inefficient.

### 4.8. Level of Online Teaching Enthusiasm

For teachers, the level of enthusiasm for the online teaching process depends on how easily they can transmit the content of the lessons (r = 0.522, *p* < 0.01), how easily they can solve the examples proposed to students (r = 0.476, *p* < 0.01), how easily they can actively involve students in the class (r = 0.467, *p* < 0.01), how easily they can grade or verify their knowledge (r = 0.398, *p* < 0.01), and how easily they can interact with the student (r = 0.381, *p* < 0.01). Those results can be observed in Table 14. Of course, the level of enthusiasm decreases as the volume of work done after the teaching classes increases (r = −0.326, *p* < 0.01). 

The same factors affect the students’ level of enthusiasm regarding online teaching, but the correlations are weaker than those in the case of teachers.

### 4.9. Correlations between Teachers’ Age, English Language Proficiency Level, Educational Transition Note to the Digital Environment, and Other Variables

One of the effects of this paper on how the educational process was affected by the pandemic situation is the educational transition note to the digital environment. This note was collected from the respondents, following their perception of how they rate, on a scale of 1 to 10 (where 1 is the lowest grade indicating a disastrous situation and 10 is the highest grade indicating an excellent situation) the way in which they consider that the transition from the classic teaching system to the online one has been made successfully. Table 15 presents some important aspects regarding the correlations between teachers’ age, English language proficiency level, educational transition notes to the digital environment, and other variables considered in this study. As teachers age, they believe that education has not adapted very well to the digital environment (r = −0.237, *p* < 0.01) and that students who are not physically supervised do not have time to answer questions and solve exercises proposed until the end of online classes (r = −0.216, *p* < 0.01), which is why they do not necessarily consider that online education will be introduced into the traditional education system (r = −0.243, *p* < 0.01). In addition, older teachers are not enthusiastic about the online teaching process (r = −0.260, *p* < 0.01), one of the possible causes being the fact that as they get older and their level of use of technology in everyday life decreases (r = −0.542, *p* < 0.01). From the teachers’ point of view, the educational transition note to the digital environment is influenced by the way in which they manage to fit in with the transmission of information (r = 0.417, *p* < 0.01), making students also understand and solve what they have to do during online classes (r = 0.529, *p* < 0.01), their enthusiasm level (r = 0.467, *p* < 0.01), but also their satisfaction level regarding the interaction with the educational platform (r = 0.445, *p* < 0.01).

Teachers’ level of language skills can be an important factor that influences the online teaching process. For example, the better they know English, the more they may have access to international resources that they could use to improve their courses and to pass on various information to students (r = 0.331, *p* < 0.01), to give more examples, or to have higher demands in solving the students’ requirements (r = 0.336, *p* < 0.01). 

### 4.10. Correlations between Students’ Age, English Language Proficiency Level, Educational Transition Note to the Digital Environment, and Other Variables

Furthermore, Table 16 presents some important aspects regarding the correlations between students’ age, English language proficiency level, educational transition notes to the digital environment, and other variables considered in this study. The age of the students is an important element that influences the way the online educational process takes place. The older the students, the more mature they are, the less they are tempted to use other applications during online classes (r = −0.407, *p* < 0.01), and the less they are affected by household chores (r = −0.318, *p* < 0.01), as their level of attention to classes is not affected. In addition, their maturity positively affects the way in which they dedicate themselves to the online learning process, managing to fit in with solving the exercises in the proposed time (r = 0.254, *p* < 0.01), appreciating favorably the interaction with the teachers in the online environment (r = 0.354, *p* < 0.01). On the other hand, students who are interested in gaining higher grades, tend to use their English knowledge to find online more information about the subject discussed in classes and tend to spend more time looking for information, which highlights their inability to fit in the time allotted within the class to complete certain tasks (r = −0.252, *p* < 0.01).

From the students’ point of view, the educational transition note to the digital environment is influenced by the way in which they manage to fit in with the transmission of information (r = 0.491, *p* < 0.01), solve what they have to do during online classes (r = 0.403, *p* < 0.01), their enthusiasm level (r = 0.557, *p* < 0.01), but also their satisfaction level regarding the ease with which they can interact with teachers (r = 0.383, *p* < 0.01) or other students (r = 0.325, *p* < 0.01). Moreover, from students’ perspective, the transition to online education is negatively influenced by the level of impairment of information transmission/reception due to technical problems (r = −0.256, *p* < 0.01), level of impairment of attention to classes due to household activities (r = −0.305, *p* < 0.01), but also the level of temptation to use various applications that are not related to teaching classes during their development (r = −0.373, *p* < 0.01).

## 5. Discussion

COVID-19 has totally changed the perspective of higher education, which was mostly based on face-to-face interactivity. By analyzing both the results of other researchers facing the process of educational learning in an online environment and the output of questionnaire respondents, five main educational directions were highlighted with all their implications. Advantages and disadvantages brought by online learning were presented along with the authors’ personal experience.

1. The most used educational platform in online teaching

Online platforms were used even before the pandemic offset of COVID-19, but the global crisis has boosted the need to use daily educational e-learning platforms. The change in the intensity of the usage of educational platform (Table 2), the need to participate in courses or trainings (Figure 1), the level of English language proficiency needs for the platforms (Table 3), and the association between language and courses (Table 4 and Table 5) are parameters involved in the change from face-to-face teaching to online. All these and the type of the electronic devises (Figure 2 and Table 6) ensure the success in all the changes required by the COVID-19 pandemic. The shift from traditional learning to a completely new one, the digital environment, had several implications. To develop materials and share them both ways—teachers to students and students to teachers—several platforms were used depending on the University resources. This fact is also sustained by a study paper [31] without mentioning which ones. According to studies [22,24], the most used platforms are WhatsApp and the Microsoft Office suite. In Romania, the present research revealed that the most used platforms are Moodle, Microsoft Teams, Google Classroom, and Zoom (Table 7). Compared to other platforms, Moodle which is the most used one with 43.4% of users being students and 28.23% teachers has several features: upload files like courses and documentation, assignments, different types of quizzes, catalogue, and chat. Microsoft Teams has also gained popularity with 46.7% of users being students and 49.41% teachers due to its following features: form groups and send messages that are visible to all members of that group in real-time, elaborate quizzes and assignments, post documents, personal chat, video meetings or calls, calendar, and integrated apps like Forms, Power Apps, and Stocks. Moodle can be connected to Microsoft Teams which is a huge advantage in terms of interoperability. Following these two platforms comes Google Classroom with 16.4% of users being students and 14.11% teachers. It has all the Google suite integrated and was designed to streamline the process of sharing files. Through its facilities, teachers can evaluate each student’s progress and assign grades. From the personal experience of authors, Microsoft wants to extend the number of apps available, and interconnection of different educational platforms used for higher education to reach an improved transformation of digital education.

2. The most used strategies in online teaching

The frequent use of online platforms for teaching purposes led to an improvement of teachers’ digital skills, disregarding their age. Based on their newly developed digital skills, different strategies for the educational process in the online environment appeared. The traditional methods and strategies were replaced by new ones that better fit the online educational environment and have the aim to capture the students’ attention and be efficient in terms of knowledge transfer. These new methods of online teaching were a must during the whole COVID-19 offset and is also stated in a study [38]. Most of them focus on engaging students and try making the lessons interactive. These strategies are analyzed by the authors from both sides: teachers and students for courses, labs, and assignments materials (Table 12). The most used ones are Video animations, virtual simulations (32% by teachers, 21.3% by students), Debates, brainstorming (20% by teachers, 11.3% by students), Contests and attention to detail tests (16% by teachers, 12.5% by students). According to this research, only 12.5% of the students consider them inefficient. From a personal point of view, discussion engagement of students has a positive impact on their level to gain new knowledge, parameter highlighted also through the students’ participation degree [33]. Another strategy that had positive feedback from students consisted in inviting at a certain number of courses established by the teacher a socio-economic specialist in the field of the course to share his/her experience. There were cases in which the courses were only taught, without having electronic support provided by teachers, which led to dissatisfaction and low involvement of students. Closely connected to the teaching strategies are the disciplines that should be studied online (Table 11) because their difficulty and specificity require us to adapt.

3. How do teachers and students evaluate individual and teamwork

Based on the study developed by [20], interactivity plays a key role in e-learning. It is harder to communicate through an online platform, without seeing the facial expressions of humans and even harder to make them express their opinion towards different subjects. When it comes to online exams the best students had to suffer and the higher grades were obtained by the ones who did not get very good grades during the academic year [37]. In this study, teachers claim that students better perform individually and not in teams. On the other side, students like to work in teams (Figure 3). It should be taken into consideration that in a team sometimes the work is not divided equally and to obtain the desired output until the deadline some of the team members must assign more time and effort. This may conduct to a poor gain of knowledge of some compared to others, reflected also on their grades since the teacher is not aware of each team member’s effort. From personal experience, when it comes to online education platforms it is preferred that each student to be evaluated individually for a better assessment.

4. The daily average time spent before and after the COVID-19 pandemic to preparation/learning process

When referring to the average time spent daily for teaching and learning activities, the efforts of teachers have increased, and the ones of students have decreased (Table 8 and Table 9). This statement covered by the authors is also sustained by [27,29,33]. The amount of homework and lack of materials, which imply time to be properly elaborated, have a negative contribution on students and their level of involvement. The importance of having proper materials for online learning is also supported by [40], since many of HEIs have focused on technical issues and infrastructure.

A teacher’s activity can be easily monitored to verify if students’ support materials were uploaded on the designated online platforms established by each university. This led to an increase in time spent to develop the entire educational process. Along with this, students were overloaded with homework to meet the conditions of each discipline, therefore the quality of their assessments has decreased. The increased number of assignments was also an output to university management verifying the process towards the fulfilment of online activity. Some have adopted the elaboration of monthly activity reports. Furthermore, the time spend in online and the changes in the time allocated for classes is connected to the use of faculties website before and during the COVID-19 pandemic (Table 10), because the need information could be taken from online and not the notice board in each faculty. All shifted to online, thus the entire process is closely connected to different parameters.

5. Perception of both teachers and students towards e-learning

The perception towards e-learning triggered the student’s interest [24] but had an impact on their social communication skills [34]. The level of stress and anxiety has increased in the online environment due to many assignments [26,32], which may leave marks when it comes to the psychological well-being of students. This anxiety may also come from fear and caring for close people [41] and developed countries tried to find solutions to support students [39]. Even if they have better participation in courses and labs, they get easily bored [33] and their concentration is reduced [23]. The way in which classes are perceived in Romanian universities is exposed in Table 13. Most of them complain that classes are tiresome and boring. The fact that classes are tiresome is also sustained by teachers along with the impersonal classes. 

Moreover, the correlations between teachers’ and students age, English language proficiency level, educational transition note to the digital environment and other variables were analyzed due to that fact that these parameters have different ways of adapting to change, being able to implement strategies, use the available technology in the online process, manage to get through the online barrier with the information and be able to keep being interested in the educational process overall (Table 15 and Table 16).

We all started reluctantly to develop the e-learning processes and all the implications that come with them. The learning process in this environment required time and digital skills development. However, the adaptation of both teachers and students was fast, each assuming their own role. Due to this, there are situations in which both parties (teachers and students) prefer to carry out the activity using online educational platforms. For some people, it is easier to express themselves in a familiar environment and participate in activities from the comfort of their homes. As a downside, more students try to do more activities at the same time: work and studies, which led to a poor level of involvement and reduced performance on their tasks. Since all the activities were carried out in the online environment, the socio-relations between students and students as well as teachers and students [22], the development of interpersonal skills, including the knowledge of the university environment (campus, classrooms, library, canteen, gym, etc.) were diminished.

## 6. Conclusions

The occurrence of the COVID-19 pandemic has completely changed the way activities are conducted in all areas, even in the educational environment. This paper presents an overview from the perspective of the two parties directly involved in the educational process in the university environment (students and teachers), offering a complex perspective aimed at five main directions: the most used educational platform in online teaching; the most used strategies in online teaching; how do teachers and students evaluate individual and teamwork; the daily average time spent before and after the COVID-19 pandemic to preparation/learning process; perception of both teachers and students towards e-learning.

From a theoretical point of view, this paper contributes to the enrichment of the literature on the university educational process during the pandemic, providing evidence from Romania, but also clarifications on the perspective of their key internal stakeholders on online educational processes in higher education units. In addition, the paper highlights the emphasis that universities have placed on certain educational platforms, as well as on the teaching strategies used in the online environment. The paper also highlights that the pandemic has significantly increased the workload for both teachers and students, which has negatively impacted the level of enthusiasm for participation in the educational process, the perception of classes and subjects, and the knowledge gained by students.

From a practical point of view, the online education system should be deployed considering the needs and expectations of key internal stakeholders. This paper identifies and investigates the strategies most used in the educational teaching process, focusing on those that would work best for students so that they no longer perceive the tiring and especially boring classes. Thus, the results of this study emphasize the need for financial investments that universities should make in terms of purchasing licenses to create virtual animations or simulations for certain classes, as well as training teachers in their use. The paper also focuses on the use of techniques such as debates and brainstorming sessions, which engage students in the educational process, maintaining their attention and interest in the topics addressed, while stimulating their creativity. For an improvement of the educational process carried out in the online environment and implicitly for the development and successful adaptation of a hybrid teaching system, both parties directly involved in this process (teachers and students) should be more involved and actively participate in the classes.

Moreover, it is necessary to conduct future studies related to the factors that influence the most important directions that characterize the online learning process (such as e-learning platforms, online teaching strategies; inputs and outputs for academic performance, perception of online teaching). This pandemic was an important starting point in adapting the educational process to technological advances, which should continue to be successfully implemented in education, even after the end of the pandemic.

## Figures and Tables

**Table 1:** Demographic data.

Variables	Values	Students		Teachers	
		Frequency	Percentage	Frequency	Percentage
Gender	Male	508	60.2	52	41.26
	Female	336	39.8	74	58.74
	Total	844	100.0	126	100.0
University profile	Technical	560	66.4	78	61.9
	Economic	118	14.0	16	12.7
	Construction	18	2.1	8	6.3
	Medicine	10	1.2	10	7.9
	Law	0	0	10	7.9
	Others	138	16.4	4	3.2
	Total	844	100.0	126	100.0
Students’ age	18–19	140	16.6	-	-
	20–21	360	42.7	-	-
	22–23	156	18.5	-	-
	24–25	30	3.5	-	-
	More than 26	158	18.7	-	-
Teaching degree	Assistant professor	-	-	34	26.98
	Lecturer	-	-	46	36.51
	Associate professor	-	-	24	19.05
	Professor	-	-	22	17.46

**Table 2:** The extent to which educational platforms were used before the pandemic to prepare/conduct teaching classes.

Values	Frequency	Percent	Valid Percent	Cumulative Percent
To a very small extent	52	41.3	41.3	41.3
To a small extent	30	23.8	23.8	65.1
To a medium extent	30	23.8	23.8	88.9
To a large extent	8	6.3	6.3	95.2
To a very large extent	6	4.8	4.8	100.0
Total	126	100.0	100.0	

**Table 3:** Participation in courses or trainings related to the use of online platforms depending on the level of English language proficiency.

The Level of English Language Proficiency	Teachers			Students		
	No	Yes	Total	No	Yes	Total
A1	2	6	8	46	20	66
A2	2	6	8	50	22	72
B1	20	14	34	146	22	168
B2	22	22	44	278	70	348
C1	4	14	18	104	20	124
C2	4	10	14	36	16	52
Total	54	72	126	660	170	830

**Table 4:** Chi-Square results for the associations between participation in courses or trainings related to the use of online platforms and the teachers’ and students’ level of English.

	Teachers			Students		
Observed Values	Value	df	Asymp. Sig. (2-Sided)	Value	df	Asymp. Sig. (2-Sided)
Pearson Chi-Square	10.836 a	5	0.055	18.873 b	5	0.002
Likelihood Ratio	11.211	5	0.047	18.457	5	0.002
Linear-by-Linear Association	0.709	1	0.400	1.258	1	0.262
N of Valid Cases	126			830		

a. 4 cells (33.3%) have an expected count of less than 5. The minimum expected count is 3.43. b. 0 cells (0.0%) have an expected count of less than 5. The minimum expected count is 10.65.

**Table 5:** Chi-Square results for the associations between participation in courses or trainings related to the use of online platforms and the students’ level of digital skills.

Observed Values	Value	df	Asymp. Sig. (2-Sided)
Pearson Chi-Square	10.115 a	3	0.018
Likelihood Ratio	9.212	3	0.027
Linear-by-Linear Association	5.605	1	0.018
N of Valid Cases	830		

a. 0 cells (0.0%) have an expected count of less than 5. The minimum expected count is 9.01.

**Table 6:** Type of electronic devices used by students in online classes.

Values	Frequency	Percent	Valid Percent	Cumulative Percent
Desktop	110	13.0	13.0	13.0
Laptop	550	65.2	65.2	78.2
Tablet	8	.9	0.9	79.1
Smartphone	176	20.9	20.9	100.0
Total	844	100.0	100.0	

**Table 7:** The most used educational platforms in online teaching.

Educational platforms	Students%	Teachers%
Moodle	43.4	28.23
Microsoft Teams	46.7	49.41
Zoom	8.8	8.23
Google Classroom	16.4	14.11
Google Meet	6.6	0
Others	0.7	0

**Table 8:** Correlations between the average daily duration allocated before and after the pandemic to the preparation/teaching process, after the end of online classes, for students and teachers.

Observed Values	Students	Teachers
Pearson Correlation	0.439 **	0.338 **
Sig. (2-tailed)	0	0
N	824	126

Note: **. Correlation is significant at the 0.01 level (2-tailed).

**Table 9:** Average daily duration allocated before the pandemic to the preparation/teaching process, after the end of online classes.

Values	Teachers %		Students%	
	Before COVID-19	After COVID-19	Before COVID-19	After COVID-19
Less than 2 h	69.8	7.9	41.2	30.0
Between 2 and 4 h	23.8	50.8	49.9	44.8
Between 4 and 6 h	4.8	31.7	6.8	19.6
More than 6 h	1.6	9.5	2.2	5.6
Total	100	100	100	100

**Table 10:** Using the faculty website before and during the COVID-19 pandemic.

Observed Values	Teachers	Students
Pearson Correlation	0.425 **	0.163 **
Sig. (2-tailed)	0.000	0.000
N	126	828

Note: **. Correlation is significant at the 0.01 level (2-tailed).

**Table 11:** Identifying the most difficult disciplines to be studied online from students’ and teachers’ perspective.

Disciplines That Are Difficult to Be Studied Online	Teachers%	Students%
Disciplines involving exact mathematical and technical sciences	60.9	52.9
Disciplines involving practical activities and presence in laboratories	17.4	25.4
Disciplines related to the medical field	10.9	0
Disciplines that involve more the vocational part	2.2	5.7
Disciplines that previously did not involve technology at all	2.2	0
All disciplines	6.5	9
All disciplines can be easily studied in the online environment	0	7

**Table 12:** The most useful online teaching strategies.

Strategies	Teachers%	Students%
Video animations, virtual simulations	32	21.3
Debates, brainstorming	20	11.3
Contests and attention to detail tests	16	12.5
Platforms for real-time use of student progress	16	8
Returning to classical education	8	12.5
Team projects	8	9.4
Graphic diagrams, summaries	0	9.4
Providing more practical examples	0	8.1
Using graphics tablets so that teachers could draw diagrams at the same time with students	0	7.5

**Table 13:** The way online classes are perceived.

The Way Online Classes Are Perceived	Students%	Teachers%
Impersonal classes	18.2	38.1
Pleasant classes	28	14.3
Engaging classes	14	6.3
Boring classes	28.4	3.2
Tiresome classes	49.8	25.4
Ingenious classes	10.9	7.9
Inefficient classes	0	3.2
Useful classes	0	1.6

**Table 14:** Correlations between teachers’ and students’ level of online teaching enthusiasm and other variables.

Variables	Info.Time	Response.Time	E.Cont.Tr	E.Solve	E.Active	E.Verify	E.Grading	E.Int.Stud	E.Int.Col	E.Work.Dclasses	E.Work.Aclasses
TLE	0.425 **	0.422 **	0.522 **	0.476 **	0.467 **	0.398 **	0.437 **	0.381 **	0.192 *	−0.195 *	−0.326 **
SLE	0.259 **	0.271 **	0.440 **	0.393 **	0.388 **	0.363 **	0.315 **	0.323 **	0.289 **	0.078 *	0.017

Note: **. Correlation is significant at the 0.01 level (2-tailed); *. Correlation is significant at the 0.05 level (2-tailed); r- Pearson Coefficient; TLE—teachers’ level of online teaching enthusiasm online; SLE—students’ level of online teaching enthusiasm; Info.Time—transmitting the information related to the teaching hours in the allocated time, without exceeding their period; Response.Time—students’ response time to the proposed questions during the time allocated to the teaching hours, without exceeding their period; E.cont.tr—ease of content transmission; E.solv—ease in solving examples; E.active—ease in being able to actively participate in class; E.verify—ease in verifying the knowledge gained by students; E.grading—ease of grading during classes; E.int.stud—ease of interaction with the students; E.int.col—ease of interaction with colleagues; E.work.dclasses—ease in the workload submitted during classes; E.work.aclasses—ease in the workload submitted after classes.

**Table 15:** Correlations between teachers’ age, English language proficiency level, educational transition note to the digital environment, and other variables.

Variables	Age	English Language Proficiency Level	Educational Transition Notes to the Digital Environment
English language proficiency level	−0.349 **	1	
Note related to the educational transition to the digital environment	−0.237 **	0.088	1
Volume of information of the prepared theoretical support	0.071	0.046	−0.157
Volume of student requirements	−0.088	0.344 **	0.104
Number of exercises performed	−0.068	0.129	−0.197 *
Number of topics developed	−0.049	−0.063	−0.153
Transmission of information related to online classes in the allocated time, without exceeding their period	−0.179 *	0.098	0.417 **
Time in which the students answer the proposed questions in the time allocated to the online classes, without exceeding their period	−0.216 *	0.003	0.529 **
Ease in transmitting content	−0.090	0.331 **	0.172
Ease in solving examples	−0.109	0.336 **	0.172
Ease in being able to actively participate in class	−0.026	0.113	0.157
Ease in verifying the knowledge gained by students	0.007	0.107	0.350 **
Ease of grading during classes	0.030	0.101	0.374 **
Ease of interaction with the students	−0.016	0.030	0.278 **
Ease of interaction with colleagues	0.072	−0.084	0.118
Ease in the workload submitted during online classes	0.064	0.067	−0.139
Ease in the workload submitted after online classes	0.205 *	−0.076	−0.177 *
Level of enthusiasm regarding online teaching	−0.260 **	0.177 *	0.476 **
Level of impairment of information transmission/reception due to technical problems	0.050	−0.297 **	−0.082
Level of impairment of attention to classes due to household activities	0.133	−0.192 *	−0.183 *
Level of use of the faculty’s website before the pandemic	−0.055	0	0.234 **
Level of use of the faculty website during the pandemic	0.020	−0.313 **	0.104
Note for class interaction with students	−0.140	0.140	0.258 **
Level of use of technology in daily activities	−0.542 **	0.357 **	0.262 **
Level of interaction with the educational platform	−0.254 **	0.162	0.445 **
The extent to which educational platforms were used before the pandemic to prepare/conduct teaching classes	−0.324 **	0.306 **	0.355 **
The extent to which educational platforms are used to prepare/conduct teaching hours after the onset of the pandemic	−0.221 *	0.099	0.031
Average daily duration allocated before the pandemic to the preparation/teaching process, after the end of teaching classes	−0.122	0.273 **	0.131
Average daily duration allocated in the context of the pandemic of the preparation/teaching process, after the end of the online classes	0.151	0.113	0.103
The extent to which online education will be introduced into the traditional education system	−0.243 **	−0.170	0.423 **

Note: * Correlation is significant at the 0.05 level (2-tailed). ** Correlation is significant at the 0.01 level (2-tailed).

**Table 16:** Correlations between students’ age, English language proficiency level, educational transition note to the digital environment, and other variables.

Variables	Age	English Language Proficiency Level	Educational Transition Notes to the Digital Environment
English language proficiency level	−0.281 **	1	
Note related to the educational transition to the digital environment	0.218 **	−0.130 **	1
Volume of information of the prepared theoretical support	0.024	0.034	0.292 **
Volume of student requirements	−0.149 **	0.163 **	−0.104 **
Number of exercises performed	0.055	0.084 *	0.115 **
Number of homework required by teachers	−0.030	0.121 **	−0.098 **
Transmission of information related to online classes in the allocated time, without exceeding their period	0.254 **	−0.245 **	0.400 **
Time in which the students answer the proposed questions in the time allocated to the online classes, without exceeding their period	0.314 **	−0.252 **	0.374 **
Ease in transmitting content	0.208 **	−0.010	0.491 **
Ease in solving problems	0.119 **	0.014	0.403 **
Ease in being able to actively participate in class	0.069 *	0.053	0.369 **
Ease in verifying the knowledge gained	0.114 **	−0.066	0.372 **
Ease of grading during classes	0.053	−0.031	0.361 **
Ease of interaction with the teachers	0.148 **	−0.021	0.383 **
Ease of interaction with colleagues	0.117 **	−0.173 **	0.325 **
Ease in the workload submitted during online classes	−0.093 **	0.052	0.044
Ease in the workload submitted after online classes	−0.068	0.080 *	0.01
Level of enthusiasm regarding online teaching	0.267 **	−0.062	0.557 **
Level of impairment of information transmission / reception due to technical problems	−0.225 **	0.102 **	−0.256 **
Level of impairment of attention to classes due to household activities	−0.318 **	0.130 **	−0.305 **
Level of temptation to use various applications that are not related to teaching classes during their development	−0.407 **	0.261 **	−0.373 **
Level of use of the faculty’s website before the pandemic	0.028	0.067	0.085 *
Level of use of the faculty website during the pandemic	−0.112 **	0.043	−0.008
Note for class interaction with teachers	0.354 **	−0.110 **	0.357 **
Level of use of technology in daily activities	−0.175 **	0.200 **	0.057
Level of interaction with the educational platform	0.040	0.062	0.234 **
The extent to which online education will be introduced into the traditional education system	0.151 **	0.007	0.299 **

Note: * Correlation is significant at the 0.05 level (2-tailed). ** Correlation is significant at the 0.01 level (2-tailed).

## Data Availability

Not applicable.
